# Beyond "Lack of Political Will": Elaborating Political Economy Concepts to Advance "Thinking and Working Politically": Comment on "Health Coverage and Financial Protection in Uganda: A Political Economy Perspective"

**DOI:** 10.34172/ijhpm.2022.7297

**Published:** 2022-05-22

**Authors:** Aloysius Ssennyonjo

**Affiliations:** ^1^Department of Health Policy Planning and Management, Makerere University School of Public Health, Kampala, Uganda.; ^2^Department of Public Health, Institute of Tropical Medicine, Antwerp, Belgium.; ^3^Institute of Development Policy (IOB), University of Antwerp, Antwerp, Belgium.

**Keywords:** Politics, Ideas, Institutions, Universal Health Coverage, Health Reforms, Power

## Abstract

Political economy analysis (PEA) has been advanced as critical to understanding the political dimensions of policy change processes. However, political economy (PE) is not a theory on its own but draws on several concepts. Nannini et al, in concert with other scholars, emphasise that politics is characterised by conflict, contestation and negotiation over interests, ideas and power as various agents attempt to influence their context. This commentary reflects how Nannini et al wrestled with these PEA concepts - summarised in their conceptual framework used for PEA of the Ugandan case study on financial risk protection reforms. The central premise is that a common understanding of the PEA concepts (mainly structure-agency interactions, ideas, interests, institutions and power) forms a basis for strategies to advance *thinking and working politically*. Consequently, I generate several insights into how we can promote politically informed approaches to designing, implementing and evaluating policy reforms and development efforts.

## Introduction

 There is an emerging consensus within the global community that politics matters in policy and development practice. In fact, in many instances, the lack of traction in the policy process has been explained in terms of ‘*lack of political will.’*^1^ However, such thinking has been rightly critiqued as simplistic^1^ and a lazy excuse for failure to perform political analysis.^2^ Hence, the increased rallying around the mantra- ‘*thinking and working politically.’*^2^ However, defining politics and related concepts is not straightforward.^3^

 Nannini et al,^4^ in their paper on health financing reforms in Uganda, underscore the relevance of the political economy (PE) lens in understanding the dynamics of advancing universal health coverage aspirations. The authors applied a conceptual framework with several domains, including stakeholders and institutions, politics (interests and ideologies), policy implementation and outcomes. In this commentary, I advance that thinking and working politically starts with understanding, defining well and usefully differentiating the key PE analytical concepts.

 Hudson and Leftwich’s^2^ definition of politics spotlights the difficulties in unpacking this concept. Accordingly, politics can be understood as the interaction of the

 “*…structures, institutions and operation of power and how it is used in the competition, conflict and deliberation over ideas, interests, values and preferences; where different individuals, groups, organisations and coalitions contest or cooperate over resources, rights, public rules and duties, and self-interest; where deals are struck, and alliances made or broken; and where establishing, maintaining or transforming political settlements, institutions and policies is an ongoing process” *(p. 5). 

 From this complex but comprehensive definition, we can delineate that the political process is characterised by the (1) complex operation and interaction of different forms and sources of power and (2) influence of structures, ideas, institutions and interests^5^. However, one glaring observation is that Nannini et al started with a broad definition of politics only to reduce politics to two concepts (ideologies and interests). The rest of this paper attempts to refocus on the broad conception of politics by unpacking the notions of structure-agency relations, ideas, interests, institutions and power.

## Structure-Agency Interactions

 Nannini’s framework rightly gives actors (labelled stakeholders and institutions) a central role in their political economy analysis (PEA). However, we cannot delink PEA from the *‘structure-agency’ *debates in social sciences that question the extent to which the observed outcomes (eg, change in behaviour, policies and actions) result from the context or actions of strategic actors.^2,6^ PEA explores the dynamic interaction between context and conduct. Nannini and colleagues^4^ demonstrated the actors and their respective influence through various actions in good detail. For example, they indicate how political actors supported user fees abolition in Uganda during an election cycle. At the same time, they highlighted how some actors (eg, employers) have actively opposed the introduction of the National Health Insurance Scheme (NHIS) in Uganda. Arguably, one of the most exciting parts is the “Politics for Health Financing” section, where these interactions between actors and the environment were analysed. They demonstrated how the PEA framework can help illuminate how agents (individuals, groups or organisations) act, considering the constraints and opportunities deriving from their local and broader conditions. To reinforce this logic, the following assertions grounded in social science theory are vital.^2^ First, the structure does not determine but shapes behaviours. Second, whereas agents work politically (make strategic decisions) to pursue their ideas and interests, they do so in a space of limited possibilities. Put simply, the structure is the medium for agency. Without structures, agents cannot act, and without agents, structures cease to exist.^2^ Understanding the bidirectional interaction between structure and agency is a precondition to working politically.

## Ideas, Interests and Institutions

 Structures are material and social, constructed from shared *ideas*.^2^ Agents have to interpret the opportunities and risks facing them, often in a situation of uncertainty. Exploring agents’ beliefs, values, and other cognitive filters is central to understanding how they act. Yet, ideational analysis tends to miss in many PEAs.^7^ Therefore, one strength of the Nannini et al is the consideration of ideational factors as major explanatory variables in PEA. For example, the authors revealed that health financing reforms in Uganda were contingent on the ideologies of actors about free healthcare and the neoliberal logic of market supremacy promoted by the global players like the World Bank.^4^ However, the paper falls short of elaborating on the concept of ideas and how they shape(d) action. This could be due to space issues, but it is prudent to reflect more on this matter.

 To support ideational analysis in PEA, understanding how ideas are defined or classified is intuitive. For instance, Hudson and Leftwich^2^ categorised ideas into (a) universally and collectively held beliefs such as religion that shape the social world or (b) normatively held views of what is right and wrong, including ideas of how the world does or should work. Relatedly, Beland ^8^ described three types of ideational processes; (a) ideas as the interpretation of the issues and policy problems, (b) ideas as assumptions that guide the selection of alternative policy solutions and (c) ideas as “framings” to justify policy direction and reason for the change. In other words, ideas shape how policy problems and solutions are understood.

 Another PEA concept explored by Nannini et al is *interests*. Interests are often presented in relation to ideational analysis.^3^ Nannini and colleagues’ arguments tend to follow conventional views that interests generate ideas to legitimate and justify them.^2^ However, we need to recognise counterarguments that agents use ideas and interpretations to construct their interests.^8^ Thus, ideas help develop and frame interests for or against policy choices. Interests are real but not objective. They are rather subjective and emerge from complex and conditional assessment of possibilities of success under prevailing context based on reflective and strategic reasoning.^2^ One major strength of Nannini et al is a thick description of how different actors reacted differently to the same context. Consequently, we can infer how they interpreted and weighed up their opportunities and constraints. However, such analysis could be enriched by exploring the evolutions of actors’ interests as these are not static over time.

 Relatedly, Nannini et al also demonstrate that the focus on interests in the policy process needs to transcend policy adoption of policy goals toward implementation (domain c). I make a subtle but essential observation. Whereas actors might agree on the ultimate policy goal (ie, financial risk protection), the interest in specific tools (eg, free healthcare or NHIS) is variable, contingent and contentious. Policy scholars argue that policy change gets down to the choice of instruments.^10^ Unsurprisingly, the interests in the goals pursued through NHIS were trumped by perceptions of political risks and threats to the political interests of influential policymakers. Working politically warrants remembering that agents perceive and respond differently to incentives inherent in the focal policy. They “don’t bend like reeds in the wind.”^2^ Hence, understanding the variability and volatility of actors’ interests can help explain why there is little support for specific instruments (NHIS) despite evidence of the agreement on the overall policy aspirations (financial risk protection).

 Similar to ideas and interests, *“institutions”* are central to PEA. However, Nannini et al conflate institutions and organisations as actors. This approach is strongly critiqued by institutionalists such as Scott,^11^ who argue that these are very distinct concepts. Institutions are the formal or informal rules of the game that constrain or facilitate human action.^1^ As part of the structural configurations, institutions require “*institutional work*” to be established and maintained.^3,12^ Without political work, they degenerate. Institutional arrangements are modified or maintained through processes of power and political work. Therefore, those applying PEA must pay attention to “processes and activities that produce, reproduce, change institutions and the resources that sustain them”^11^ (p. 57).

 It is further essential to underscore that institutions are social facts. So, ideas about the institutions are as important as the institutions themselves. Moreover, ideas and institutions interact in complex and dynamic ways to shape the behaviour and decisions of policy actors.^2^ One political dimension of institutions is that they are not neutral. They disadvantage some actors while creating advantages for others. Following Hudson and Leftwich,^2^ I underscore two critical considerations when deploying PEA. First, identifying and describing the existing institutional context of the focal policy. Second, interrogating how the institutions shape what actors can and cannot do and how they provide resources for agents to act and shape change dynamics and context.

## Centrality of Power

 One significant limitation in Nannini and colleagues’ paper is the failure to explicitly examine the notions of *power*, yet, power is the *sine qua non* in political action and analysis.^13^ As the preceding paragraphs indicate, power emanates from different sources, such as ideas. Power is the “channel and mechanism through which structures ‘do’ structuring”^2^ (p. 77). Power and its distribution, forms and expression- is the force through which other actors’ ideas, beliefs and interests are influenced and the political context in which agents act is shaped.^2^ In brief, how power is conceptualised and related to other concepts in PEA needs further interrogation.

 One of the commonest political actions is strategic framing to influence how other agents interpret their realities and how they should or could act in them.^1^ This observation is linked to the notion of ideational power - “the capacity of actors (whether individual or collective) to influence other actors’ normative and cognitive beliefs through applying ideational elements”^9^ (p. 322). Examining the various sources and mechanisms of ideational power is vital to advancing thinking and working politically. Yet, the authors do not pursue this angle profoundly. I propose that the typology by Carstensen and Schmidt^9^ could be instructive in exploring ideational power ( see Box 1 below).

**Box 1. Typology of Ideational Power**

*Power through ideas*: Capacity of actors to persuade other actors to accept and adopt their views of what to think and do through the use of ideational elements.

*Power over ideas*: Capacity of actors to control and dominate the meaning of ideas. Manifests as (a) actors with the power imposing their ideas; (b) powerless actors seeking to compel other actors to conform with their ideas or norms; (c) actors having the capacity to resist even considering alternative ideas.

*Power in ideas*: Focuses on background ideational processes – constituted by systems of knowledge, discursive practices and institutional setups such as epistemic communities – that in important ways affect which ideas enjoy authority at the expense of others

Source: Carstensen and Schmidt 2016.^9^

Power is embedded in, framed and shaped by institutions and agents in a bi-directional interplay leading to “institutional politics” of a reform situation.^14^ To extend these insights, Lawrence proposes three dimensions of institutional politics,^14^ namely (a) institutional control, (b) institutional agency, and (c) institutional resistance. Institutional control refers to the effect of institutions on the beliefs, actions and behaviours of individual or collective actors. This is related to the notion of institutional power and “power in ideas” discussed above. Here power is often subtle but demonstrated by actors’ compliance with rules and norms.^14^ Institutional agency refers to the observable work of actors to create, modify or disrupt institutions.

 In contrast, institutional resistance relates to the attempts of actors to resist both institutional agency and control. According to the above conception, power can be categorised into structural or agential forms that underpin institutional control and institutional agency or resistance^2^. In line with the structure-agency discussion above, the power of agents draws on institutional and structural power. Structural power is embedded and expressed in institutions and can be considered systemic. In contrast, agential power is episodic and characterised by actors’ discrete expression of strategic actions.^12,14^

 Nannini et al^4^ affirm coalition formation as political actions to amplify agential power and effectively wrestle with entrenched interests and power relationships. Their paper also spotlights manifestations of structural power embedded in bureaucratic government systems. Health financing reforms were presented to occur within a web of existing practices and rules leading to path-dependence. For instance, public financial management systems act as institutional arrangements shaping how health financing reforms like the NHIS take place.^15^ In addition, the “free healthcare” policy was perceived as inconsistent with the introduction of premiums under NHIS- counteracting policy adoption.^4^

 In conclusion, this paper raises key considerations to practically change the ‘rules of the game’ of how PEA is deployed as both a practical and theoretical tool. First, disaggregating while carefully linking the PEA concepts increases their viability as analytical tools for researchers and policymakers to think and work politically. Second, the judicious application of PEA during different phases of policy development is valuable. Practical support is required to tactically deploy PEA prospectively to anticipate and guide the political dynamics in development practice. Finally, more reflections on the practical and methodological considerations in PEA (such as the benefits and limitations of conducting PEA as ‘outsiders’ with no local co-authors as in Nannini et al) are recommended.

## Ethical issues

 Not applicable.

## Competing interests

 Author declares that he has no competing interests.

## Author’s contribution

 AS is the single author of the paper.

## Disclaimer

 The views expressed in this article are for the author and not the position of the institutions of affiliation.
